# A systematic review of mobility instruments and their measurement properties for older acute medical patients

**DOI:** 10.1186/1477-7525-6-44

**Published:** 2008-06-05

**Authors:** Natalie A de Morton, David J Berlowitz, Jennifer L Keating

**Affiliations:** 1 Department of Physiotherapy, School of Primary Health Care, Faculty of Medicine, Nursing and Health Sciences, Monash University, Australia; 2 Northern Clinical Research Centre, Northern Health, Australia

## Abstract

**Background:**

Independent mobility is a key factor in determining readiness for discharge for older patients following acute hospitalisation and has also been identified as a predictor of many important outcomes for this patient group. This review aimed to identify a physical performance instrument that is not disease specific that has the properties required to accurately measure and monitor the mobility of older medical patients in the acute hospital setting.

**Methods:**

Databases initially searched were Medline, Cinahl, Embase, Cochrane Database of Systematic Reviews and the Cochrane Central Register of Controlled Trials without language restriction or limits on year of publication until July 2005. After analysis of this yield, a second step was the systematic search of Medline, Cinahl and Embase until August 2005 for evidence of the clinical utility of each potentially suitable instrument. Reports were included in this review if instruments described had face validity for measuring from bed bound to independent levels of ambulation, the items were suitable for application in an acute hospital setting and the instrument required observation (rather than self-report) of physical performance. Evidence of the clinical utility of each potentially suitable instrument was considered if data on measurement properties were reported.

**Results:**

Three instruments, the Elderly Mobility Scale (EMS), Hierarchical Assessment of Balance and Mobility (HABAM) and the Physical Performance Mobility Examination (PPME) were identified as potentially relevant. Clinimetric evaluation indicated that the HABAM has the most desirable properties of these three instruments. However, the HABAM has the limitation of a ceiling effect in an older acute medical patient population and reliability and minimally clinically important difference (MCID) estimates have not been reported for the Rasch refined HABAM. These limitations support the proposal that a new mobility instrument is required for older acute medical patients.

**Conclusion:**

No existing instrument has the properties required to accurately measure and monitor mobility of older acute medical patients.

## Background

The functional independence of older people is an important indicator of their health status. Diminished independence in hospitalised older people is associated with increased risk of transfer to nursing home, carer burden, mortality and healthcare costs after discharge [1]. Independent mobility is also a key factor in determining readiness for discharge for older hospitalised patients. An instrument that accurately measures and monitors this important construct for hospitalised older patients would have a range of useful applications in clinical care.

Mobility is the focus of the Timed Up and Go (TUG) [2] and Functional Ambulation Classification (FAC) [3] and a subsection of the Barthel Index (BI) [4-6]. These instruments have limitations for measuring mobility in acutely hospitalised patients or others who exhibit a broad spectrum of ability such as community dwelling older people [7-11]. The FAC is a relatively insensitive measure of change for older acute medical patients [11]. The TUG and the BI have inadequate scale width [7-11] and do not adequately capture changes in physical health for people whose limitations are either severe or relatively modest. The TUG has a floor effect with approximately one-quarter of hospitalised older people unable to complete this test because they are too weak [9]. The BI has a ceiling effect with approximately one quarter of patients scoring within the error margin of the highest score [9]. It has also been argued that the BI is a multidimensional scale (i.e. measures multiple constructs) and consequently summation of BI item scores to obtain a total score does not yield an interpretable index [8].

Many trials in aged care in the acute hospital setting have been confounded by inadequate physical outcomes measures. The importance of measures of physical ability across the spectrum of ability has been argued by those prescribing exercise for older people [12]. Pressure on already limited healthcare resources is predicted to increase as the average population age rises. An outcome measure that can accurately measure mobility is required to identify interventions that optimize physical outcomes of hospitalised older patients and facilitate effective targeting of healthcare services.

When selecting an outcome measure for a particular clinical purpose, there are many factors to consider [13]. No systematic review assists clinicians to determine the most appropriate mobility outcome measure for older general medical patients in the acute care setting. Therefore, the aims of this review were to:

- identify potentially relevant instruments for measuring mobility in older acute medical patients.

- summarise and compare the relevant clinimetric properties of the included instruments.

## Methods

This review was conducted in two phases. Initially, a broad systematic search was performed to identify existing instruments for measuring the mobility of hospitalised older acute medical patients. For each instrument that was included, a second search was conducted to identify papers reporting research into its clinimetric properties. This second phase of searching was not constrained to studies of older patients. Data on the clinimetric properties of identified instruments were subsequently extracted and compared.

### Phase One: instrument search

#### Inclusion and exclusion criteria

Reports were included in this review if they described instruments with face validity for measuring from bed bound to independent levels of ambulation and the items were suitable for testing in an acute care hospital (e.g. did not require a laboratory or large open spaces, were not community-based tests such as transferring in and out of a car). The instrument had to be administered by observation of physical performance to counter assessment limitations associated with cognitive deficits and recall bias in hospitalised older patients. For instruments that measured across multiple domains, the report was included if a subtotal for mobility could be determined. Instrument use in the acute hospital setting is also likely to be influenced by practical factors such as the time required for test administration. Therefore this review aimed to identify an instrument that could be conducted, if necessary, during a hospital medical ward round. Based on this criterion, instruments that took greater than 10 minutes to administer, on average, were excluded. Instruments were also excluded if they were not freeware or required expensive equipment as cost is likely to be a barrier to clinical use in many acute hospital settings. Since health care providers can also vary from new graduates to experienced and specialised clinicians, it is also important that an appropriate mobility instrument does not require a minimum level of clinical experience to administer and can therefore be applied by all clinical staff. Therefore, instruments were excluded from the review if a report stipulated that a minimum level of clinical experience was required to administer the test. Instruments that were condition specific (e.g. stroke), consisted of only one item or, due to a known ceiling effect on the BI, the ambulatory items (i.e. high level items) were the same as the ambulatory items on the BI were also excluded from this review.

#### Instrument identification and selection

Electronic databases were searched without language restriction or limits on year of publication until July 2005. A sensitive search was conducted for key search terms for 'older adults', 'mobility' and 'outcome measures'. Search terms for 'older adults' and 'mobility' were limited to the title or abstract to constrain the magnitude of the review yield to a manageable size. The complete search strategy is shown in Appendix 1. Databases searched were Medline, Cinahl, Embase, Cochrane Database of Systematic Reviews and the Cochrane Central Register of Controlled Trials. All papers were screened for mobility instruments that were reported in the title or abstract. Mobility was defined according the World Health Organisation's International Classification of Functioning (ICF) [14]. Hard copies were obtained of the instruments reported in included papers.

Additional papers were identified by searching the American Physical Therapy Association Catalog of Tests and Measures [15], the UK Chartered Society of Physiotherapy website [16] and the Australian Physiotherapy Association Neurology Special Group Handbook [17]. Two independent reviewers examined hard copies of all included papers and applied inclusion and exclusion criteria. Disagreement between assessors was resolved with discussion.

### Phase Two: clinimetric search

In phase one a finite set of relevant instruments were identified. A second systematic search was then conducted to identify what was known about the clinimetric properties of each instrument. The search strategy is shown in Appendix 2. Medline, Cinahl and Embase were searched until August 2005. Papers were screened based on title and abstract for data on clinimetric properties of relevant instruments. Hard copies of potentially relevant papers were obtained. If a reason for instrument exclusion (criteria described for the phase one search) became apparent while examining clinimetric reports, the instrument was excluded.

Inclusion criteria for phase two were that data were provided on clinimetric properties of instruments identified in phase one and that these data enabled estimation of properties such as reliability, validity, minimally clinically important difference (MCID), responsiveness to change, internal structure/dimensionality or acceptability or feasibility.

### Instrument evaluation

Data were extracted for each instrument identified by this review and were summarised under each of the following categories:

#### Instrument characteristics

The instrument items, response options, scoring system, equipment requirements, time to administer and floor and ceiling effects were extracted.

#### Internal structure and dimensionality

Data reporting the results of Rasch analysis, factor analysis or Cronbach's alpha were extracted.

#### Reliability

The following data about reliability of instruments were extracted: the type of reliability study conducted (e.g. inter or intra-rater reliability), the methods employed to conduct the study (e.g. independent assessments or video recording of the same patient assessment), assessor training and the characteristics of the patient group. Reliability estimates are reported using many indices. Any of the following were extracted: intraclass correlation coefficient (ICC), Pearson's r, Spearman's rho, Bland and Altman's limits of agreement [18], the minimal detectable change with 90% (MDC_90_) or 95% (MDC_95_) confidence intervals, the root mean square of the residuals (RMS) associated with the test-retest regression or the standard error of measurement (SEM). If reliability data were not reported in the units of measurement, the SEM and MDC_90 _were calculated from related statistics where possible.

#### Validity

Reports of the opinions of experts in the field regarding instrument items or item content were extracted as evidence of face or content validity respectively. Correlational data and associated 95% confidence intervals (e.g. ICCs, Pearson's r, Spearman's rho) were extracted as evidence of convergent (high correlation with measures of related constructs) and discriminant validity (low correlation with measures of unrelated constructs). For groups of patients who are known to differ in their mobility, group mean scores (and standard deviations) and between groups comparison data were extracted as evidence of 'known groups' validity. Data that indicated a relationship between mobility instrument scores and subsequent relevant health outcomes (e.g. a regression model) were extracted as evidence of predictive validity.

#### Minimally clinically important difference

The MCID has been defined by Jaeschke, Singer and Guyatt [19] as "the smallest difference in score in the domain of interest which patients perceive as beneficial......". The MCID provides clinicians with the change in scores that patients perceive to represent an important amount of change. MCID point estimates and associated 95% confidence intervals were extracted from relevant papers. In the absence of reports that provided MCID data, the MCID was estimated using the distribution-based approach recommended by Norman et al. [20].

#### Responsiveness to change

For instruments included in this review, responsiveness indices and associated 95% confidence intervals were extracted. Data reporting significant change scores between assessments in a group of patients who were expected to change was considered adequate evidence of instrument responsiveness to change and was therefore extracted.

#### Acceptability and feasibility

Relevant data were extracted from any study that formally investigated the acceptability and/or feasibility of an instrument included in this review.

## Results

### Phase one: instrument search

The search identified 4100 papers. After screening of title/abstract, 3775 papers were excluded. From the remaining 325 papers, 178 assessment measures were identified (see Additional file 1) and hard copies were obtained. Predetermined inclusion and exclusion were applied. Seven physical performance mobility measures were included in this review:

• Clinical Outcomes Variable Scale (COVS) [21]

• Elderly Mobility Scale (EMS) [22]

• General Motor Function Assessment Scale [23]

• Goal Attainment Scale [24,25]

• Hierarchical Assessment of Balance and Mobility (HABAM) [26,27]

• Physical Disability Index [28]

• Physical Performance and Mobility Examination [29]

#### Phase two: clinimetric search

After obtaining hard copies of papers that reported the clinimetric properties of the seven remaining instruments, a further four instruments were excluded. Table 1 shows that three instruments were excluded due to a reported average administration time of more than 10 minutes. One instrument was excluded as a minimum of 1 year of clinical experience and 7 hours of training were required to administer the instrument.

**Table 1:** Reason for exclusion of mobility assessment instruments

**Instrument**	**Reason for exclusion**
**Goal Attainment Scale**	Requires a minimum of 1 year of clinical experience and 7 hours of training to administer [17].
**The Clinical Outcomes Variable Scale**	Approximately 30 minutes to administer [17].
**The General Motor Function Assessment Scale**	Average time to administer of 18 mins (range 5 to 40 mins) [23].
**Physical Disability Index**	Average time to administer of 60+/-21 minutes (range 46 – 83) [28].

**Figure 1:**
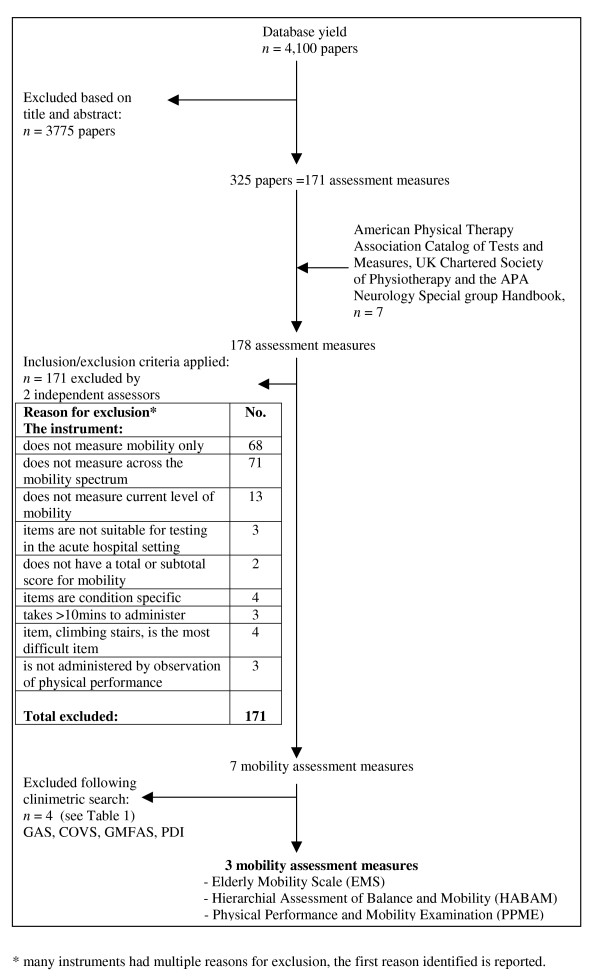
Flow diagram of process of outcome measure inclusion and exclusion.

**Figure 2:**
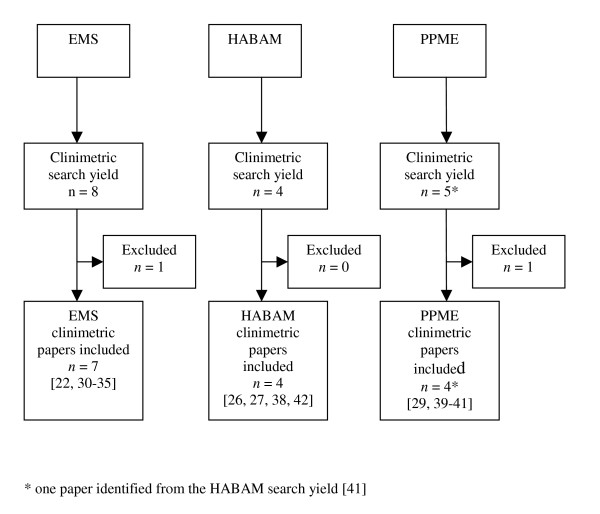
Flow diagram of clinimetric paper inclusion and exclusion.

Three instruments were included in this review and were subjected to rigorous clinimetric evaluation: the Elderly Mobility Scale (EMS) [22], the Hierarchical Assessment of Balance and Mobility (HABAM) [26,27] and the Physical Performance Mobility Examination (PPME) [29]. Figure 1 shows a flow diagram of the inclusion and exclusion of instruments in this review (Phase 1). The most common reasons for instrument exclusion were that the items did not measure across the mobility spectrum or that the instrument items measured domains other than mobility. No instrument was excluded due to cost only. For each instrument that was included, Figure 2 shows a flow diagram of the inclusion and exclusion of papers reporting the clinimetric properties of each instrument (Phase 2).

### Elderly Mobility Scale

#### Characteristics

The EMS was developed in the 1990s in England as a mobility assessment tool for frail older adults [22]. The characteristics of the EMS are summarised in Table 2. A ceiling effect has been identified for the EMS. For community dwelling older adults who had experienced a single fall in the previous 6 months, "approximately 50% of single fallers scored 19 – 20" [30] and for twenty healthy 81 to 90 year old women, all scored the highest possible score of 20 on the EMS [22].

**Table 2:** Characteristics of the EMS, HABAM and PPME

	EMS	HABAM	PPME
**Versions**	1. Original [22].	1. Original [26,42] 2. Rasch refined [27]	1. Original [29]
**Number of items**	Seven	1. 27 in the original version 2. 22 in the modified version	1. Six items
**Content**	Lying to sitting, sitting to lying, sit to stand, stand, gait, timed walk (6 meters), functional reach.	MOBILITY: bedfast, chairfast, 2 person assist +/- aid, 1 person hands on +/- aid, 1 person standby +/- aid, with aid 8–50 m, with aid > 50 m, unlimited with aid, limited 8–50 m, limited > 50 m, unlimited. TRANSFERS: total lift, 2 person assist, 1 person assist, 1 person pivot, 1 person hands-on, 1 person standby, independent with aid, independent. BALANCE: impaired static sitting, stable static sitting, stable dynamic sitting, stable static standing, stable dynamic standing, stable transfer, stable with aid, stable ambulation.	Bed mobility, transfer skills, multiple stands from chair, standing balance, step-up and ambulation.
**Time to complete**	"No more than 5 minutes" [32]	Average of 2.6 (+/- 1) minutes [41].	Approximately 10 minutes [29] 8.6 minutes (SD = 3.6 minutes) [41]
**Equipment requirements**	A bed, chair, stop watch, walking aid if necessary, a space for a standardised 6 meter walk and a functional reach test.	A bed, chair and walking aid if required.	A bed, chair, stop watch, standardised step and gait aid if required.
**Scaling method**	One response is selected by the clinician administering the test for the 7 mobility tasks. Two items are scored from 0 – 2, four items are scored from 0 – 3 and one item from 0 – 4.	The original version of the HABAM is an ordinal measure. Interval level data is provided by the Rasch converted version of the HABAM.	The PPME has two scaling methods. The pass-fail PPME provides 2 response options (pass or fail) and the 3 level PPME provides 3 response options for each item (high pass, low pass or fail). Each response option is clearly defined [29].
**Scoring**	Each item score is summed to provide a total possible score from 0 to the maximum score of 20 which represents independent mobility. Scores under 10 are considered to represent "dependence in mobility manoeuvres", 10 – 13 to indicate "borderline in terms of safe mobility" and 14 or more to be "likely to be independent in mobility" [22].	The original version of the HABAM has a total score range of 0 – 24. One point is scored for each increment in ability. Higher scores indicate higher levels of mobility. The Rasch converted HABAM has a broader interval score range of 0 to 26. A score is listed next to each item on the HABAM. Harder items have higher scores. The highest score obtained across the 3 sections of the HABAM represents the HABAM interval score. Higher scores indicate higher levels of mobility.	The pass-fail PPME provides a dichotomous scoring system for the 6 PPME items. Zero is scored for a fail. One point is scored for successfully completing each item. Items sum to obtain a maximum score of 6. In the 3 level PPME scoring system, zero is scored for a fail, one point for a low pass and two points for a high pass. The total score range is 0 – 12.
**Floor and ceiling effects**	A ceiling effect was identified for community dwelling older adults who had experienced a single fall in the previous 6 months, "approximately 50% of single fallers scored 19 – 20" [30]. Twenty healthy 81 to 90 year old women all scored the highest possible score of 20 on the EMS [22].	A ceiling effect was identified in an older acute medical patient population. Approximately 25% of patients scored the maximum possible score at hospital admission [27].	An absence of floor and ceiling effects has been reported for the 3 level scoring system [29].

#### Internal structure and dimensionality

Data on the internal consistency or unidimensionality of the EMS has not been reported.

The EMS was reported by its developer to provide ordinal level data [22].

#### Reliability

Three studies have investigated the inter-rater reliability [22,31,32] and one study has investigated the intra-rater reliability of the EMS [31]. Extracted reliability data are reported in Table 3. None of these studies reported the SEM or MDC_90 _nor provided the data required to calculate these indices. No reports provided details regarding assessor training with the EMS prior to the reliability study.

**Table 3:** Inter-rater and intra-rater reliability for the EMS

**Author**	**Population and test procedures**	**Reliability data provided**
*Inter-rater reliability*		

Smith [22]	15 inpatients or day hospital patients, 78 to 93 years were independently assessed by two assessors.	Inadequate data provided to estimate reliability.
Prosser et al. [32]	19 older acute medical patients aged 71 to 91 years, independently assessed by two assessors. Assessors were blinded to the other assessor scores.	Spearman's correlation coefficient between assessor scores, r = 0.88, p < 0.0001.
Cuijpers et al. [31]	A video recorded assessment of 28 hospitalised frail older patients rated by two independent assessors (Dutch version of the EMS). Patient age was not provided in the English abstract.	Inter-rater reliability ICC 0.95 – 0.97 (p value not provided in the published abstract).* Bland and Altman limit of agreement of 3 points.

*Intra-rater reliability*		

Cuijpers et al. [31]	A video recorded assessment of 28 hospitalised frail older patients rated by two independent assessors (Dutch version of the EMS). Patient age was not provided in the English abstract.	Intra-rater reliability ICC 0.97 (p value not provided in the published abstract).* Bland and Altman limit of agreement = 3 points.

ICC = intraclass correlation coefficient

* the type of ICC employed was not reported

#### Validity

The EMS items and response options are worded clearly and simply and the seven items can be classified as measuring the domain of mobility. Although the qualitative methods employed to develop the EMS items were not clearly reported by the test developer [22], item generation and development based on expert opinion and the existing literature provides evidence of face and content validity.

Convergent validity was reported in two studies. Smith [22] reported that EMS scores were highly correlated with BI scores (Spearman's rho = 0.96) and Functional Independence Measure scores (Spearman's rho = 0.95) for 36 inpatients/day hospital patients aged 70 – 93 years. The statistical significance of these correlations was not reported. Similarly, Prosser and Canby [32] reported a significant and high correlation between EMS and BI scores (r = 0.79, p < 0.001) for 66 patients aged 66 – 96 years admitted to hospital with an acute medical illness.

Evidence of known groups validity for the EMS was obtained from three studies [22,30,32]. Smith [22] reported that 20 healthy older adults scored 20 points (the maximum score) on the EMS compared to 36 people with mobility deficits who had a median score of 9 (range 0 – 20). Smith also reported higher EMS scores for hospitalised patients who were discharged to home (range 14 – 20 points) compared to those discharged to home with a carer (range 5 – 13 points) or discharged to nursing home (range 0 – 6 points). Between group differences were not formally tested in this study but group scores were likely to have been significantly different based on the range of reported scores. Prosser and Canby [32] reported similar group differences in discharge destination data and significant between group differences (p < 0.001) were confirmed with a chi squared test in this study.

Evidence of known groups validity for the EMS was also reported by Chiu et al. [30]. Community dwelling older persons with multiple falls in the six months prior to the study scored significantly lower on the EMS compared to older persons who had experienced no falls or only a single fall in the six months prior to the study (p < 0.001).

Spilg et al. [33] reported a statistically significant relationship between EMS scores at discharge from a geriatric day hospital (*n *= 76 patients with mobility problems) and the risk of two or more falls during a four month follow up period (logistic regression, p = 0.008). These data demonstrated evidence of predictive validity for the EMS.

#### Minimally clinically important difference

No studies reported the MCID for the EMS. However, two studies [30,34] provided data that allowed the MCID to be estimated using the recommendations of Norman et al. [20]. The MCID for the EMS was approximately 2 points or 10% the scale width.

#### Responsiveness

Only one study investigated the responsiveness to change of the EMS [35]. Eighty three percent of patients in a falls rehabilitation program who were expected to improve in their mobility improved on EMS scores compared to 42% on BI scores and 35% on Functional Ambulation Classification scores [35]. A significant improvement in EMS scores was identified between assessments (p < 0.001). This provides evidence that changes in EMS scores reflect changes in patients who are expected to change.

#### Acceptability and feasibility

No formal study of acceptability or feasibility has been reported. Prosser and Canby [32] reported that the EMS was easy to apply in an older acute medical population. They implied that familiarisation with test procedures was required, but provided no detail.

### Hierarchical Assessment of Balance and Mobility

#### Characteristics

The HABAM was developed in the 1990's in Canada [26]. The HABAM was developed to evaluate balance and mobility for older patients admitted to hospital with a medical illness. A summary of the characteristics of the HABAM are reported in Table 2. A ceiling effect was identified for the HABAM in an older acute medical patient population. Approximately 25% of patients scored the maximum possible score at hospital admission [27].

#### Internal structure and dimensionality

MacKnight and Rockwood [27] investigated the internal consistency and unidimensionality of the HABAM with data collected from 204 older people who were admitted to hospital with a medical illness. Based on the results of this study, the HABAM appears to be an internally consistent scale.

MacKnight and Rockwood [27] conducted principal components analysis and identified four factors with eigenvalues greater than one (13.86, 4.02, 1.85 and 1.15). The four components accounted for 51%, 15%, 7% and 4% of the total scale variance respectively. All of the HABAM items loaded on the first component. Rasch analysis of the same data confirmed the unidimensionality of the HABAM after the removal of six items. The HABAM therefore appears to measure one construct and provide interval level data. However, data supporting the overall fit of the data to the Rasch model were not provided in the published report. In addition, data for 53 of the 204 people were extreme because these persons successfully completed all items [27]. This indicates a ceiling effect of approximately 26% for the HABAM on the Rasch converted logit scale.

In the same study, the three sections of the HABAM, mobility, transfers and balance, each had high correlation with the HABAM total score and with each other [27]. Cronbach's alpha for the HABAM total score, mobility, transfers and balance subscales were reported to be 0.97, 0.92, 0.92 and 0.88 respectively. These are all higher than the alpha value of 0.80 that is commonly considered acceptable [36]. This indicates high inter-item correlation and thus high internal consistency of the HABAM. However, a Cronbach's alpha value that is greater than 0.90 is also reported to represent high levels of item redundancy [37]. Therefore, the HABAM may consist of items that provide similar mobility challenges.

#### Reliability

The inter-rater reliability for ordinal raw scores on the original HABAM was examined on 15 patients aged 65 years or older admitted to a general medicine or geriatric assessment unit [26]. Each patient was independently assessed by two researchers and a high correlation (ICC = 0.94) was reported between assessor scores. The type of ICC, the MDC_90 _and the SEM were not provided in the published report. However, the baseline standard deviation of HABAM raw scores for 28 patients (that included the 15 patients in the reliability study) was reported. This standard deviation was employed to estimate a SEM and a MDC_90 _of 2.2 and 5.1 points respectively. This MDC_90 _is high as it represents approximately 20% of the HABAM scale width. The reliability of the Rasch refined HABAM has not been published.

#### Validity

Face validity for the HABAM was obtained by an experienced person in geriatric medicine assessing the instrument items during its development. The HABAM items appear to be a hierarchical list of mobility challenges ranked conceptually from easy to hard. Items range from the easiest item, *needs positioning in bed*, to the hardest item, *unlimited mobility*. Evidence of content validity for the HABAM was obtained by the data fitting the Rasch model and thus indicating that the HABAM is a unidimensional measure of mobility.

Two studies have provided evidence of convergent validity for the original version of the HABAM [26,38] by reporting a high correlation between HABAM scores and measures of related constructs. A Spearman's rank correlation of 0.76 between HABAM and BI change scores was reported for an older acute medical patient population [26] and 0.69 for a nursing home population [38]. A Spearman's rank correlation of 0.74 was identified between HABAM and BI motor subscale change scores for an older acute medical inpatient population [26]. A definition of the mobility subscale was not provided in the published report but the mobility items presumably include walking, transfers and stairs.

Evidence of discriminant validity for the original HABAM was identified by low correlations between HABAM scores and measures of other constructs. In an older acute medical patient population, a low correlation was identified between HABAM change scores and the Mini Mental State Examination (Spearman's rank = 0.15), Instrumental Activities of Daily Living (Spearman's rank = 0.30) and the Spitzer Quality of Life Scale change scores (Spearman's rank = 0.39) [26]. In a nursing home patient population, HABAM change scores had low correlation with change scores for the Goal Attainment Scale (Spearman's rank = 0.17), Cumulative Illness Rating Scale (Spearman's rank = -0.32) and the Brief Cognitive Rating Scale (Spearman's rank = -0.04) [38]. No evidence of known groups validity has been reported.

#### Minimally clinically important difference

The MCID for the HABAM has not been investigated in a published report. However, using Norman et al.'s [20] recommendations, the MCID was estimated to be 4.5 points for the original version of the HABAM using the very similar baseline standard deviations provided in reports by MacKnight and Rockwood [26] and Gordon et al. [38].

#### Responsiveness

The responsiveness to change of the original HABAM has been investigated in two studies using both the Effect Size Index and the Relative Efficiency Index [26,38]. For measurements recorded at hospital admission and discharge in an older acute medical population, the HABAM had an Effect Size Index of 0.59 compared to 0.35 and 0.51 for the BI and BI mobility subscale respectively [26]. In the same study, the Relative Efficiency Index for the HABAM was reported to be approximately three times greater than for the BI. In a nursing home population, the HABAM was found to be more responsive to change than the BI but less responsive to change than the Goal Attainment Scale using both the Effect Size Index and Relative Efficiency Index [38]. However, neither of these reports [26,38] provided confidence intervals for these responsiveness indices. It remains unclear if statistically significant differences exist between these point estimates of responsiveness.

#### Acceptability and feasibility

MacKnight and Rockwood (2002) conducted a study that investigated the acceptability and feasibility of the HABAM. In a sample of 19 hospitalised older medical patients, 89% of patients reported that the HABAM testing procedure did not bother them in any way and 100% of patients reported that they would not mind performing the HABAM test daily. Twenty-six staff were also interviewed after administering the HABAM. Of these staff, 77% reported that the HABAM provides useful information and 46% reported that they could incorporate the HABAM into their daily hospital rounds.

### Physical Performance and Mobility Examination

#### Characteristics

The PPME was designed in the USA in the 1990s to measure physical functioning and mobility for hospitalised older adults [29]. The characteristics of the PPME are shown in Table 2. An absence of floor and ceiling effects has been reported for the 3 level scoring system [29].

#### Internal structure and dimensionality

No studies have investigated the internal structure or dimensionality of the PPME.

#### Reliability

Two reports were found about the intra-rater reliability of the PPME [29,39] and one report of the inter-rater reliability [29]. Although none of these studies provided reliability estimates in the units of measurement, the MDC_90 _was estimated from the data provided in the published reports. Extracted and derived reliability data are shown in Table 4.

**Table 4:** Reliability data for the PPME

**Author**	**Population and test procedures**	**Scoring System**	**ICC (95%CI)**	**Standard deviation (SD)**	**SEM**	**MDC_90_**
*Intra-rater reliability*						

Winograd et al. [29]	50 hospitalised patients, mean age 74.8 (SD = 7.9). Tested 48 hours apart. If the patient reported or the chart indicated a change in condition, the patient was excluded. This study included 33 patients.	Pass-fail scoring system.	0.99*	Pooled SD not provided. Baseline SD 2.1 for sample 1 (*n *= 146) and 1.7 for sample 2 (*n *= 352). Weighted average SD = 1.8.	0.18	0.42
Winograd et al. [29]	As above.	3 level scoring system.	0.98^#^	Pooled SD not provided. Baseline SD 2.8 for sample 1 (*n *= 146) and 3.1 for sample 2 (*n *= 352). Weighted average SD = 3.0.	0.42	0.97
Sherrington and Lord [39]	Test retest of 30 older people, mean age 81.1 years (SD = 7.5) following hip fracture (16 rehabilitation hospital inpatients and 14 community dwelling). Two assessments one day apart.	3 level scoring system.	0.96^# ^(0.92 -0.98)	Test 1 SD = 2.4 and test 2 SD = 2.2. Weighted average SD = 2.3.	0.46	1.07

*Inter-rater reliability*						

Winograd et al. [29]	31 patients, mean age 75 (SD = 6.43), selected from (1) acute medical unit inpatients that had impaired mobility and (2) acute medical and surgical inpatients aged ≥ 65 years. Two assessors independently rated each patient's performance on the PPME.	Pass-fail scoring system.	0.99	Pooled SD not provided. Baseline SD 2.1 for sample 1 (*n *= 146) and 1.7 for sample 2 (*n *= 352). Weighted average SD = 1.8.	0.18	0.42
Winograd et al. [29]	As above.	3 level scoring system.	0.99	Pooled SD not provided. Baseline SD 2.8 for sample 1 (*n *= 146) and 3.1 for sample 2 (*n *= 352). Weighted average SD = 3.0.	0.3	0.7

SD = standard deviation, ICC = intraclass correlation coefficient

* Phi coefficient, ^#^ICC (3,1)

#### Validity

The PPME has face and content validity for measuring mobility based on expert opinion (group interviews with physical therapists) and existing instruments employed to develop the PPME [29].

Data extracted as evidence of convergent and discriminant validity for the PPME are shown in Table 5. Convergent validity for the PPME was identified by a significant and high correlation between PPME scores and other measures of physical function. Discriminant validity was indicated by a low correlation between PPME scores and measures of cognitive and emotional status. Confidence bands were not provided for these point estimates. No evidence of known groups validity has been reported.

**Table 5:** Validity data for the PPME

**Author**	**Patient population (≥ 65 years)**	***n***	**2 level scoring system**	**3 level scoring system**
*Convergent validity*			*Total PPME score correlation with:*	*Total PPME scores correlate highly with:*

Winograd et al. [29]	Older patients hospitalised with mobility impairment	88	Self reported physical functioning and mobility scores, r = 0.61, p < 0.001.	Self reported physical functioning and mobility scores, r = 0.73, p < 0.001.
Winograd et al. [29]	Hospitalised older medical and surgical patients	154	Self reported physical functioning and mobility scores, r = 0.71, p < 0.001.	Self reported physical functioning and mobility scores, r = 0.77, p < 0.001.
	Hospitalised older medical and surgical patients	154	ADL scores, r = 0.70, p < 0.001.	ADL scores, r = 0.68, p < 0.001.

*Discriminant validity*			*Total PPME score correlation with:*	*Total scores have low correlation with:*

Winograd et al. [29]	Older patients hospitalised with mobility impairment	97	MMSE scores, r = 0.36, p < 0.001.	MMSE scores, r = 0.38, p < 0.001.
Winograd et al. [29]	Hospitalised older medical and surgical patients	154	MMSE scores, r = 0.36, p < 0.001.	MMSE scores, r = 0.38, p < 0.001.
	Hospitalised older medical and surgical patients	86	Geriatric depression scores, r = 0.23, p < 0.001.	Geriatric depression scores, r = 0.28, p < 0.001.

#### Minimally clinically important difference

The MCID has not been reported for the PPME. Using Norman et al.'s [20] recommendations, the MCID was estimated. Based on data reported by Winograd et al. [29], the MCID was calculated to be 0.9 for the dichotomous PPME scoring system. Based on data reported in three studies [29,39,40] the MCID was calculated to range from 1.15 to 2.15 for the 3 level PPME scoring system.

#### Responsiveness

No reports of the responsiveness to change of the PPME were identified.

#### Acceptability and feasibility

MacKnight et al. [41] reported the acceptability and feasibility of the PPME in a sample of 19 hospitalised older medical patients. Eighty-nine percent of patients reported not being bothered by the PPME test and no patients reported any objection when asked if they would mind performing this test everyday. Twenty-six medical staff were interviewed after administering the PPME and 76.9% reported that the PPME provided useful information. However, staff reported being unable to incorporate the PPME into their daily rounds.

### Comparison of error estimates and clinically important change

Table 6 shows the estimated measurement error and MCID for the EMS, HABAM and PPME scores. The limit of agreement is a more conservative estimate of measurement error than the MDC_90_. The MDC_90 _and limit of agreement provide an estimate of the minimum change score required to be 90% and 95% confident respectively that measurement error has been overcome. Measurement error appears to be greater than the MCID for the EMS and the original version of the HABAM but not for the PPME. These data were not available for the Rasch refined version of the HABAM.

**Table 6:** MDC_90 _and MCID estimates for the EMS, HABAM and PPME

	**MDC_90_**	**MDC_90 _% of scale width**	**MCID**	**MCID % of scale width**
**EMS (0 – 20)**	3*	15.0%	2	10.0%
HABAM** (0 – 24)	5.1	21.3%	4.5	18.8%
**PPME (0 – 6)**	0.42	7%	0.9	15.0%
**PPME (0 – 12)**	0.7 – 1.07	5.8% – 8.9%	1.15 – 2.15	9.6% – 17.9%

* Bland and Altman 'limit of agreement' [18], MDC_90 _could not be estimated for the EMS.

** original version of the HABAM. Data not available for the Rasch refined HABAM.

## Discussion

This review identified a plethora of outcome measures that have been employed to measure activity limitation for older adults. However, only three suitable instruments, the EMS, HABAM and PPME were found for measuring and monitoring changes in mobility for older people. Clinimetric evaluation identified that each of these instruments has significant limitations.

Older acute medical patients have a very broad range of physical abilities [7,9-11]. For this reason they are a difficult patient group to measure on one scale. Tests that are developed in hospitalised populations, such as the Barthel Index, typically have a ceiling effect in an older acute medical population as there are no items to challenge the subgroup whom are independently ambulant [7-11]. Tests that are developed in community populations, such as the TUG, typically have a floor effect in an older acute medical population as a proportion of these patients cannot stand [7,9-11].

In the acute hospital setting, the physical and cognitive ability of older patients can also fluctuate over short time periods. It is therefore likely that direct examination of performance is required to provide the most accurate indication of ability. Many instruments identified in this review were designed for administration by self report. Designing a physical performance test that covers a broad spectrum of abilities and is quick and easy to administer in the acute hospital setting poses a challenging task for test developers. The difficulty of this challenge is reflected in the large number of outcome measures that were identified in this review but do not have the properties required for clinical application in this patient group.

Although differing methods were employed to develop the EMS, HABAM and PPME, each of these instruments consists of bed transfers, chair transfers, balance and walking items. However, the item wording, testing protocols and scoring systems vary considerably across instruments. For example, for bed mobility tasks, the EMS provides a three-point response option for patient independence with transfers from *lying to sitting *and *sitting to lying*. The HABAM provides a dichotomous response option for *positions self in bed *and *lying to sitting independently *and the PPME assesses *sitting up in bed (from lying down) *using either a two or three option scoring system.

Based on the World Health Organisation's International Classification of Functioning (ICF) [14], the EMS, HABAM and PPME contain items that are classified under 'activity and participation' as measuring the domain of 'mobility.' Each of these instruments has face and content validity for measuring mobility. Scores on each of these measures appear to have high correlation with measures of related constructs and low correlation with measures of unrelated constructs, providing evidence of convergent and discriminant validity respectively. Evidence of known groups validity has been reported for the EMS but not for the HABAM or PPME.

Only the HABAM has been subjected to Rasch or factor analysis to investigate the dimensionality of the underlying construct. Following Rasch analysis, items were removed from the original version of the HABAM and the remaining HABAM items were reported to fit the Rasch model. This indicates that the Rasch refined HABAM is a unidimensional measure of mobility and fit of the data to the Rasch model also provides further evidence of content validity for the HABAM. The internal structure of the EMS or PPME has not been investigated and thus the validity of item score summation to obtain a total mobility score for these instruments is therefore unknown. Fit of HABAM data to the Rasch model also indicates that the Rasch converted HABAM scores provides interval compared to the ordinal level data provided by the EMS and PPME.

In a head-to-head comparison of the HABAM and the PPME in a sample of 19 hospitalised older adults, the HABAM was statistically significantly quicker to administer and rated to be feasible by a larger proportion of clinicians in the acute hospital setting. The HABAM was reported to take on average 2.6 minutes (range 1 – 4) to conduct compared to 8.6 minutes (range 3 – 16) for the PPME. Most users felt that the HABAM (92.3%) and PPME (76.9%) provided useful information. However, no staff reported being likely to include the PPME into their daily rounds compared to 46.2% for the HABAM. Although the feasibility of the EMS has not been investigated, the HABAM has fewer equipment requirements than the EMS and PPME and is therefore likely to be the more feasible of these instruments in the acute hospital setting.

An important limitation of the HABAM is the ceiling effect identified in an older acute medical population [27]. In a sample of 204 older medical patients, approximately one-quarter of patients did not fail any items. The HABAM is therefore not suitable for monitoring improvements in mobility for a significant proportion of independently ambulant older medical patients. Rasch analysis of HABAM data identified *unlimited mobility *to be the most difficult item [27]. To overcome the HABAM ceiling effect, additional high level mobility items would be required.

Error estimates are required in the units of measurement to facilitate the accurate interpretation of test scores. Neither the MDC_90 _nor the SEM were provided in published reports for the EMS, HABAM or PPME. The 'limit of agreement' recommended by Bland and Altman [18] was reported to be 3 points for the EMS in an English abstract of a Dutch publication [31]. This estimate represents 15% of the EMS scale width. The MDC_90 _was estimated from data provided in the published reports for the original HABAM and PPME. For MDC_90 _calculations for these instruments, assumptions were required to estimate the standard deviation and therefore the MDC_90 _may be greater than estimated. The MDC_90 _estimated for the HABAM represented approximately 20% of the scale width and for the PPME the MDC_90 _represented approximately 10% of the scale width regardless of the scoring system.

Although the MCID for the EMS, HABAM or PPME have not been reported, estimates indicated that a change score of greater than 2 points (10% of scale width) is likely to represent an important change in mobility for the EMS, 4 points for the HABAM (19% of scale width), 1 point for the PPME two level scoring system (9% of scale width) and 2 points for the PPME three level scoring system (16% of scale width). The confidence intervals for these MCID point estimates are not known. The MDC_90 _point estimates were greater than the MCID for the EMS and original HABAM but not for the PPME. This is a limitation of the EMS and HABAM as important change and measurement error cannot be partitioned. Neither the MDC_90 _or MCID data could be calculated for the Rasch refined HABAM.

The responsiveness to change of the EMS, HABAM and PPME has not been tested in a head-to-head comparison and therefore the relative responsiveness of these instruments is not known.

### Strengths and Limitations

This review has provided an important contribution to knowledge by providing healthcare professionals and the scientific community with a comprehensive evaluation of existing measures of activity limitation for hospitalised older acute medical patients. Other strengths of this review are that it provides a comprehensive summary of the measurement properties of the EMS, HABAM and the PPME, demonstrates methods for rigorously evaluating the clinimetric properties of health instruments, provides convincing evidence for the need to develop a new mobility outcome measure for older acute medical patients and was conducted in two phases to maximise the sensitivity of this review. Limitations of this review were that only manuscripts published in English were eligible for inclusion in this review and that some of the search terms for phase one were limited to title and abstract to constrain the magnitude of the search yield to a manageable size.

## Conclusion

This review identified that no existing instrument has all the properties required to accurately measure and monitor changes in mobility for older acute medical patients. Selecting an outcome measure that is not appropriate for a particular purpose can result in clinical trials that are confounded by inadequacy of selected measures or patient assessments that are misleading or provide information of little or no clinical utility. Three instruments were included in this review, the EMS, HABAM and PPME. Clinimetric evaluation indicated that the HABAM has the most desirable properties of the three instruments. The HABAM provides interval level data, is quick and feasible, appears to be more responsive to change than the BI and has minimal equipment requirements. However, the HABAM has the limitation of a ceiling effect in an older acute medical patient population and reliability and MCID estimates have not been reported for the Rasch refined HABAM. This review provides information about the relative merits of existing activity limitation outcome measures for hospitalised older adults and is a valuable resource for clinicians and researchers. The limitations of existing instruments supports the proposal that a new mobility instrument is required for older acute medical patients.

## Competing interests

The authors declare that they have no competing interests.

## Authors' contributions

NdM conceived and designed the review, acquired the data, analysed and interpreted the data, wrote the manuscript and has given final approval of the version to be published. DB contributed to the analysis and interpretation of the data, has been involved in the final stages of drafting of the manuscript and given approval for the version to be published. JK contributed to the conception and design of the review, the analysis and interpretation of data, drafting of the manuscript and has given final approval of the version to be published.

## Appendix 1. Medline search strategy for existing mobility outcome measures

1 Aged.ti,ab.

2 old$.ti,ab.

3 elder$.ti,ab.

4 frail.ti,ab.

5 geriatric$.ti,ab.

6 1 or 2 or 3 or 4 or 5

7 (function$ adj2 (status or decline or physical or ability)).ti,ab.

8 mobility.ti,ab.

9 (independence adj10 (physical or function$)).ti,ab.

10 (dependence adj10 (physical or function$)).ti,ab.

11 activities of daily living.ti,ab.

12 (gait or walk$ or ambulat$).ti,ab.

13 disability.ti,ab.

14 handicap.ti,ab.

15 (impairment adj2 (physical or function$)).ti,ab.

16 participation.ti,ab.

17 7 or 8 or 9 or 10 or 11 or 12 or 13 or 14 or 15 or 16

18 (functional adj5 (outcome or assessment)).ti,ab.

19 *Questionnaires/

20 *Exercise Test/

21 *Treatment Outcome/

22 *"OUTCOME AND PROCESS ASSESSMENT (HEALTH CARE)"/or *GERIATRIC ASSESSMENT/or *"PROCESS ASSESSMENT (HEALTH CARE)"/or *"OUTCOME ASSESSMENT (HEALTH CARE)"/

23 data collection/or *health surveys/or *health care surveys/

24 *"Recovery of Function"/

25 18 or 19 or 20 or 21 or 22 or 23 or 24

26 6 and 17 and 25

27 (pediatric$ or paediatric$).ti,ab.

28 child$.ti,ab.

29 27 or 28

30 26 not 29

31 limit 30 to humans

## Appendix 2. Medline search strategy for clinimetric papers of existing mobility outcome measures

1 clin?metric.mp.

2 exp PSYCHOMETRICS

3 person?metric.mp.

4 validity.mp.

5 reliability.mp.

6 unidimensional$.mp.

7 (Rasch adj analys$).mp.

8 discriminability.mp.

9 responsiveness.mp.

10 appropriateness.mp.

11 precision.mp.

12 interpretability.mp.

13 acceptability.mp.

14 practicability.mp.

15 feasibility.mp.

16 (floor adj effect).mp.

17 (ceiling adj effect).mp.

18 (minimal detectable change or MDC).mp.

19 (minimally clinically important difference or MCID).mp.

20 sensitivity.mp.

21 (standardised response mean or SRM).mp.

22 Guyatt's responsiveness statistic.mp.

23 (Effect adj size).mp.

24 1 or 2 or 4 or 5 or 6 or 7 or 8 or 9 or 10 or 11 or 12 or 13 or 14 or 15 or 16 or 17 or 18 or 19 or 20 or 21 or 22 or 23

25 **NAME OF EACH OUTCOME MEASURE. mp.**

26 24 and 25

## Supplementary Material

Additional file 1 Appendix 3. List of the 178 assessment measures identified by the initial search yield. Appendix 3. List of the 178 assessment measures identified by the initial search yield. Click here for file
