# Supplementary material for: A systematic review of mobility instruments and their measurement properties for older acute medical patients
Source: Health Qual Life Outcomes. 2008 Jun 5;6:44. doi: 10.1186/1477-7525-6-44 (PMC2430553; doi:10.1186/1477-7525-6-44)
Supplement: Additional file 1 — Appendix 3. List of the 178 assessment measures identified by the initial search yield. Appendix 3. List of the 178 assessment measures identified by the initial search yield. [file 1477-7525-6-44-S1.doc]

Appendix 3. List of the 178 assessment measures identified by the initial search yield.

|  | **Outcome Measures identified** |
| --- | --- |
| 1 | Active Australia Survey (AAS) |
| 2 | Acute Care Index of Function (ACIF) |
| 3 | Adelaide Activities Profile Questionnaire |
| 4 | ADL-Oriented Assessment of Mobility |
| 5 | AMC Linear Disability Score |
| 6 | American College of Rheumatology Functional Classification for hip OA |
| 7 | ASA Physical Status Classification |
| 8 | ASIA Impairment Scale |
| 9 | Arthritis Impact Measurement Scale |
| 10 | Assessment of Living Skills and Resources (ALSAR) |
| 11 | Austoms |
| 12 | Baecke Questionnaire |
| 13 | Barthel Index |
| 14 | Berg Balance Scale |
| 15 | Bristol ADL Scale |
| 16 | CHART |
| 17 | Chronic Respiratory Questionnaire (CRQ) |
| 18 | Chula ADL Index |
| 19 | Clinical Global Impression of Change in Physical Frailty |
| 20 | Clinical Outcomes Variable Scale |
| 21 | Comprehensive Older Persons Evaluation |
| 22 | CS-PFP |
| 23 | Deaconess Elder Care Assessment of Risk (DECAR) |
| 24 | Dijon Physical Activity Score |
| 25 | Direct Assessment of Functional Status (DAFS) |
| 26 | Disability Ratings Scale (DRS) |
| 27 | Duke Activity Status Index |
| 28 | EQ5D |
| 29 | Elderly Mobility Scale (EMS) |
| 30 | Environmental Analysis of Mobility Questionairre |
| 31 | Eortc QLQ-C30 |
| 32 | Evaluation of Dialy Activities |
| 33 | Everyday Abilities Scale for India |
| 34 | Exercise of Self-care Agency Scale |
| 35 | Falling Risk Inventory Questionnaire |
| 36 | Falls Handicap Inventory |
| 37 | Fast Evaluation of Mobility, Balance and Fear (FEMBAF) |
| 38 | Functional Assessment Measure (FAM) |
| 39 | Functional Independence Measure (FIM) |
| 40 | FIM and FAM |
| 41 | Frail Elderly Functional Assessment |
| 42 | Frenchay Activity Index |
| 43 | Fuctional Ambulation Categories (FAC) |
| 44 | Functional Ambulation Classification (FAC) |
| 45 | Fuctional Assessment Inventory (FAI) |
| 46 | Functional Assessment Screening Instrument (FASI) |
| 47 | Functional Assessment Screening Questionnaire Items (FASQ) |
| 48 | Functional Assessment Staging |
| 49 | Functional Autonomy Measurement Scale (SMAF) |
| 50 | Functional Disability Assessment Scale |
| 51 | Functional Disability Scale (FDS) |
| 52 | Functional Interference Estimate |
| 53 | Functional Performance Inventory (FPI) |
| 54 | Fullerton Functional Fitness Test (FFT) |
| 55 | Functional Limitations Profile |
| 56 | Functional Outcome of Sleep Questionnaire (FOSQ) |
| 57 | Functional Reach |
| 58 | Functional Rating Index |
| 59 | Functional Assessment Inventory |
| 60 | Functional Status Assessment (FSA) |
| 61 | Functional Status Examination |
| 62 | Functional Status Questionnaire |
| 63 | Functional Status Index (FSI) |
| 64 | General Function Score |
| 65 | Geriatric Functional Rating Scale (GFRS) |
| 66 | Geriatric Outcomes Scoring System (GOSS) |
| 67 | Geriatric Postal Screening Survey and Scoring Scale |
| 68 | Global Assessment of Functioning Scale (GAF) |
| 69 | Global Deterioration Scale (GDS) |
| 70 | Groningen Functional Capacity Test |
| 71 | Groningen Activity restriction Scale |
| 72 | General Motor Function Assessment Scale |
| 73 | Gross Motor Function Classification System Levels for Children |
| 74 | Gross Motor Function Measure |
| 75 | Goal Attainment scaling (GAS) |
| 76 | Hamrin Activity Index |
| 77 | Hierarchical Assessment of Balance and Mobility (HABAM) |
| 78 | Health Assessment Questionaire - Disability Index (HAQ-DI) |
| 79 | HiMAT |
| 80 | Historical Physical Activity Questionnaire (HPAQ) |
| 81 | Human Activity Profile (HAP) |
| 82 | Index of Life Skills |
| 83 | Instrumental Activity Measure (IAM) |
| 84 | IOWA Self Assessment Inventory |
| 85 | Karnofsky Performance Status Scale |
| 86 | Katz ADL |
| 87 | Katz Physical ADL |
| 88 | Kenny Self Care Evaluation |
| 89 | Klein Bell ADL Scale |
| 90 | Kohlman Evaluation of Living Skills (KELS) |
| 91 | Late Life Function and Disability Instrument: Disability Component |
| 92 | Late Life Function and Disability Instrument |
| 93 | Lawton IADL |
| 94 | LEIPAD |
| 95 | Leisure Time Physical Activities |
| 96 | Life Space Questionnaire |
| 97 | London Handicap Scale |
| 98 | London Psychogeriatric Scale |
| 99 | Modified Motor Assessment Scale (MMAS) |
| 100 | Motor Assessment Scale (MAS) |
| 101 | Manchester Respiratory Activities of Daily Living Questionnaire |
| 102 | Mayo Portland Adaptability Inventory (MPAI) |
| 103 | Mob H Scale |
| 104 | Mob T Scale |
| 105 | Mobility Aids Status (MAIDS) |
| 106 | Mobility-Related Physiological Limitations (MOBLI) |
| 107 | Mobility Classification Tool |
| 108 | Modified Cumulative Illness Scale |
| 109 | Medical Outcomes Study Instrument (MOSI) |
| 110 | MSAS |
| 111 | MOS SF-20 |
| 112 | Neurological Patients Fuctional Inderpendence Scale |
| 113 | New York Heart Association Classification |
| 114 | Northwestern Functional Status Review Instrument |
| 115 | Northwestern University Disability Scale |
| 116 | Obstacle Course |
| 117 | Older Americans Resources and Services (OARS) |
| 118 | Oswestry Disability Index |
| 119 | Physical Activity Scale for the Elderly (PASE) |
| 120 | Patient Evaluation Conference System (PECS) |
| 121 | Physician Estimated Mobility Score (PEMS) |
| 122 | PC PART |
| 123 | Physical Disability Index (PDI) |
| 124 | Parkinson's Disease ADL scale |
| 125 | Performance Assessment of Self Care Skills |
| 126 | Performance Orientated Assessment of Mobility |
| 127 | Performance Test of ADL |
| 128 | Psychogeriatric Rating Scale |
| 129 | Physical Performance Battery |
| 130 | Physical Performance Test Scoring Sheet |
| 131 | Physical Self Maintenance Scale |
| 132 | Physician based assessment and counselling for exercise (PACE) |
| 133 | Physiotherapy Functional Mobility Profile |
| 134 | Pulmonary Functional Status Scale (PFSS) |
| 135 | Physical Performance and Mobility Examination (PPME) |
| 136 | PULSES |
| 137 | Questionnairre of Functional Ability |
| 138 | Rankin Score |
| 139 | Rapid Disability Rating Scale |
| 140 | Restricted Activity |
| 141 | Rivermead Mobility Index (RMI) |
| 142 | Scale of Functional Capacity |
| 143 | Seattle Angina Questionnaire |
| 144 | Self Efficacy for Functional Activities Scale |
| 145 | Sensory-Oriented Mobility Assessment Instrument (SOMAI) |
| 146 | Self Efficacy for Functional Ability |
| 147 | SF-12 |
| 148 | SF-20 |
| 149 | SF-36 |
| 150 | Shanas Index of Disability |
| 151 | Sheehan Disability Scale |
| 152 | Sherbroke Postal Questionnaire |
| 153 | Short Musculoskeletal Function Assessment Questionnaire |
| 154 | Social Dependence Concepts and Performance Scores |
| 155 | Sodring Motor Evaluation of Stroke |
| 156 | Specific Activity Scale |
| 157 | Strawbridge Questionnaire |
| 158 | Standardised Practical Equipment Test |
| 159 | Stroke Adapted 30 item version of the Sickness Impact Profile (SA-SIP30) |
| 160 | Stroke Impact Scale |
| 161 | Stroke Impairment Assessment Set (SIAS) |
| 162 | Subjective Index of Physical and Social Outcome |
| 163 | Sub-Saharan Africa Activity Questionnaire (SSAAQ) |
| 164 | Summary Performance Scores (SPS) |
| 165 | Sickness Impact Profile (SIP)(adapted) |
| 166 | Six Minute Walk Test |
| 167 | Ten Meter Walk Test |
| 168 | The Medical Outcomes Study Instrument |
| 169 | The Physical Capacity Evaluation |
| 170 | Timed Manual Performance Test |
| 171 | Timed Up and Go Test (TUG) |
| 172 | Tinetti Balance Assessment Tool |
| 173 | Toglias Category Assessment (TCA) |
| 174 | Tuft's Assessment of Motor Performance |
| 175 | WHO-ILAR COPCORD Core Questionnaire |
| 176 | WHOQOL-HIV |
| 177 | WOMAC |
| 178 | Yale Activity Scale |
